# Direct oriented growth of armchair graphene nanoribbons on germanium

**DOI:** 10.1038/ncomms9006

**Published:** 2015-08-10

**Authors:** Robert M. Jacobberger, Brian Kiraly, Matthieu Fortin-Deschenes, Pierre L. Levesque, Kyle M. McElhinny, Gerald J. Brady, Richard Rojas Delgado, Susmit Singha Roy, Andrew Mannix, Max G. Lagally, Paul G. Evans, Patrick Desjardins, Richard Martel, Mark C. Hersam, Nathan P. Guisinger, Michael S. Arnold

**Affiliations:** 1 Department of Materials Science and Engineering, University of Wisconsin-Madison, Madison, Wisconsin 53706, USA; 2 Center for Nanoscale Materials, Argonne National Laboratory, Argonne, Illinois 60439, USA; 3 Department of Materials Science and Engineering, Northwestern University, Evanston, Illinois 60208, USA; 4 Department of Engineering Physics, École Polytechnique de Montréal, Montréal, Québec, Canada H3C 2A7; 5 Department of Chemistry, Université de Montréal, Montréal, Québec, Canada H3C 3JT; 6 Department of Chemistry, Northwestern University, Evanston, Illinois 60208, USA

## Abstract

Graphene can be transformed from a semimetal into a semiconductor if it is confined into nanoribbons narrower than 10 nm with controlled crystallographic orientation and well-defined armchair edges. However, the scalable synthesis of nanoribbons with this precision directly on insulating or semiconducting substrates has not been possible. Here we demonstrate the synthesis of graphene nanoribbons on Ge(001) via chemical vapour deposition. The nanoribbons are self-aligning 3° from the Ge〈110〉 directions, are self-defining with predominantly smooth armchair edges, and have tunable width to <10 nm and aspect ratio to >70. In order to realize highly anisotropic ribbons, it is critical to operate in a regime in which the growth rate in the width direction is especially slow, <5 nm h^−1^. This directional and anisotropic growth enables nanoribbon fabrication directly on conventional semiconductor wafer platforms and, therefore, promises to allow the integration of nanoribbons into future hybrid integrated circuits.

## Abstract (web summary)

Semiconducting armchair graphene nanoribbons with sub-10 nm width are of great technological importance but yet to realize. Here, the authors report growth of such nanoribbons on germanium and controlled crystallographic orientation and well-defined armchair edges are obtained.

## Graphical abstract

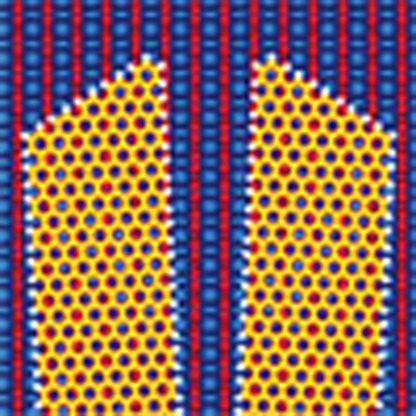

Graphene nanoribbons are excellent charge^1^ and thermal^2^ conductors that can exhibit high current-carrying capacity^3^ and novel magnetic and spin-polarized edge states^4^,^5^,^6^ depending on their crystallographic orientation, edge structure and width. Unlike continuous two-dimensional graphene, which is semimetallic, one-dimensional (1D) graphene nanoribbons can be semiconducting, allowing for substantial modulation of their conductance and enabling their application in semiconductor logic, high-frequency communication devices, optoelectronics, photonics and sensors in which a bandgap is needed to achieve high performance. Their bandgap roughly varies inversely with ribbon width and the largest bandgaps are expected for ribbons with armchair edge orientation^7^.

While achieving a ribbon width of <10 nm is necessary to induce a technologically relevant bandgap that is substantially greater than *k*_B_*T* of 25 meV at room temperature^5^,^7^, sub-10 nm resolution is beyond the limits of conventional optical and electron-beam lithography. Moreover, top-down lithographic techniques in which ribbons are etched from continuous graphene sheets result in nanostructures with relatively rough, defective edges, which lead to Coulomb blockade^8^ and localized electronic^9^,^10^ and phonon states^11^ and, consequently, degrade the high charge carrier mobility^12^,^13^,^14^ and thermal conductivity^2^ of graphene.

These deficiencies, in part, can be overcome, via bottom-up organic synthesis on metal surfaces^15^,^16^,^17^ and in solution^18^,^19^ as well as by unzipping graphite^20^ and carbon nanotubes^21^ in solution, to yield ribbons with sub-10 nm width and smooth edges. However, surface-assisted organic synthesis yields short ribbons (∼20 nm) and unzipping graphite and carbon nanotubes does not offer control over the ribbon crystallographic orientation. Moreover, the controlled placement and alignment of ribbons onto substrates from solution has proven to be difficult.

Scalable nanoribbon fabrication has also been reported via epitaxial growth on templated SiC nanofacets^22^ and by chemical vapour deposition (CVD) on surface features, such as steps^23^, twins^24^ and trenches^25^, as well as on patterned catalysts in which growth is confined to predetermined areas that define the ribbon dimensions^26^,^27^,^28^,^29^. However, with these approaches, ribbons with sub-10 nm width have not been demonstrated and the catalyst template determines the ribbon edge structure rather than a more precise self-defining growth mechanism.

Here we show that the CVD of graphene on Ge(001) can be controlled to yield oriented nanoribbons with sub-10 nm width and smooth armchair edges. Previous work on integrating graphene with Ge has primarily focused on large-area graphene monolayers. For example, continuous graphene films have been transferred onto Ge from other substrates^30^,^31^. Furthermore, continuous graphene monolayers have been grown via CVD directly on Ge(001) by Wang *et al.*^32^ and Ge(110) and Ge(111) by Lee *et al.*^33^. However, in these previous studies, nanoribbons were not observed in partial growth experiments. In this work, we report that high aspect ratio nanoribbons can be directly grown on the Ge(001) facet by tailoring the CVD conditions to maximize the anisotropy of crystal growth. It is critical to operate in a regime in which the growth rate is especially slow, <5 nm h^−1^ in the width direction. Nanoribbons are grown at atmospheric pressure using various growth temperatures (860<*T*<935 °C), CH_4_ mole fractions (3.7 × 10^−3^<
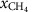
<1.6 × 10^−2^), and H_2_ mole fractions (0.17<
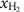
<0.33), as summarized in Supplementary Table 1. By tuning *T*, 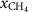, 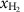 and the growth time (*t*), the growth anisotropy is tailored to yield ribbons from the bottom-up with controlled width (*w*), length (*l*) and aspect ratio. Using conditions in which the anisotropy is maximized, isolated ribbons are prevalent on the Ge(001) surface even after *t*>18 h. In contrast, Wang *et al.*^32^ used a relatively fast growth rate in which continuous graphene films were synthesized on Ge(001) in *t*<100 min.

## Results

### Growth behaviour

**Figure 1:**
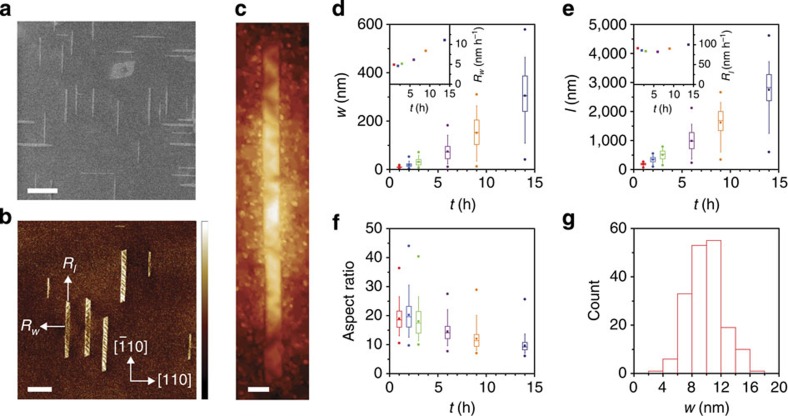
Growth evolution of graphene nanoribbons on Ge(001). (**a**–**c**) SEM (**a**), AFM phase (**b**) and STM (applied bias −2 V, current 200 pA) (**c**) images. Scale bars are 400 nm in (**a**,**b**) and 10 nm in **c**. Phase scale bar in **b** is 4.9°. (**d**–**f**) Ribbon *w* (**d**), *l* (**e**) and aspect ratio (**f**) plotted against *t*. Horizontal lines in the boxes define the 25th, 50th and 75th percentiles, whiskers indicate the 5th and 95th percentiles, circles define the range and squares give the mean. Insets of (**d**,**e**) are the mean *R*_*w*_ and *R*_*l*_, respectively, plotted against *t*. (**g**) Histogram of *w* from the 1 h growth in **d**–**f**. The ribbons in **a**,**c** are synthesized at 910 °C with 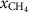 of 0.0092 and 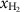 of 0.33 for 2 and 1.5 h, respectively, whereas the ribbons in **b** are grown at 910 °C with 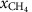 of 0.0066 and 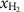 of 0.28 for 4 h. The ribbons in **d**–**g** are synthesized with the same parameters as **a**,**c** but *t* is varied between 1 and 14 h.

Several general observations are made regarding graphene growth on Ge(001) by analysing representative scanning electron microscopy (SEM), atomic force microscopy (AFM) and scanning tunnelling microscopy (STM) images in Fig. 1a–c. Following nucleation, graphene crystals evolve anisotropically, resulting in nanoribbons with high aspect ratio and smooth, straight edges. Raman spectroscopy indicates that the 2D:G ratio and the 2D peak full-width-at-half-maximum are 6.0 and 28 cm^−1^, respectively, confirming that the nanoribbons are monolayer graphene^34^ (Supplementary Fig. 1). The ribbons preferentially orient closely along the 〈110〉 directions of the Ge(001) template, resulting in two ribbon orientations that are approximately perpendicularly aligned. These two orientations exist with equal probability. For ribbons with *w*<10 nm, the short ribbon edges form 60, 90 or 120° angles with the long ribbon edges. However, for wider ribbons, only angles of 60 and 120° are observed, indicating that all edges are oriented along equivalent crystallographic directions of graphene. While as high as 90% of the graphene crystals that nucleate evolve as ribbons, more compact graphene crystals with lower aspect ratio and edges that are not aligned along Ge〈110〉 directions are also observed. Interestingly, the Ge underneath the ribbons is nanofaceted, which is studied further below.

### Growth kinetics and evolution

We quantify the growth kinetics to gain insight into the processes that determine the ribbon size and aspect ratio using constant *T* of 910 °C, 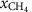 of 0.0092 and 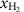 of 0.33. Both *w* and *l* increase with *t*, along with the ribbon-to-ribbon variation in *w* and *l*, which is quantified by the range of the box and whiskers in Fig. 1d,e. The mean growth rates in the *w* and *l* directions, *R*_*w*_ and *R*_*l*_, respectively, are compared in the insets of Fig. 1d,e (where *w* and *l* increase on average at twice *R*_*w*_ and *R*_*l*_). Initially, *R*_*l*_ is 90 nm h^−1^ whereas *R*_*w*_ is only 5 nm h^−1^, giving rise to the anisotropic ribbon evolution. While *R*_*l*_ is relatively constant with time, *R*_*w*_ increases to >10 nm h^−1^ after several hours. Accordingly, the mean aspect ratio decreases from 20 to 10 with increasing *t* (Fig. 1f). Stopping growth after *t* of 1 h results in ribbons with average *w* of 9.8 nm (Fig. 1g), which is below the resolution of optical and typical electron-beam lithography, and we anticipate that even narrower *w* are obtained at earlier *t*. Figure 1c shows an example of a nanoribbon with *w* of 7 nm and *l* of 160 nm.

**Figure 2:**
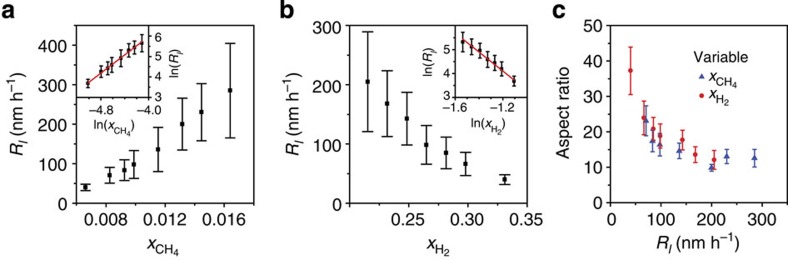
Effect of precursor composition on graphene nanoribbon growth on Ge(001). (**a**,**b**) *R*_*l*_ versus 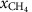 (**a**) and 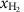 (**b**). Insets of **a**,**b** contain the same data plotted on a log–log scale with best fit line (red), which is used to find the empirical rate laws. (**c**) Aspect ratio of ribbons with *w* of 30±5 nm plotted against *R*_*l*_ for conditions in which 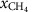 (blue triangles) and 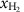 (red circles) are varied. The ribbons in **a**,**c** are grown at 910 °C with 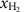 of 0.33 and 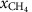 varied from 0.0066 to 0.016. The ribbons in **b**,**c** are grown at 910 °C with 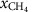 of 0.0066 and 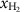 varied between 0.22 and 0.33. Error bars indicate standard deviation.

The effects of 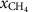, 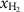 and *T* on the nanoribbon growth rate and the resulting anisotropy are also quantified. The growth rates increase as 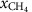 increases (Fig. 2a), 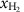 decreases (Fig. 2b), and *T* increases (Supplementary Fig. 2). Each of these parameters can be independently tuned to vary *R*_*l*_ over an order of magnitude from 30 to 300 nm h^−1^. The anisotropy varies inversely with *R*_*l*_, independent of whether 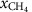 or 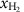 is changed (Fig. 2c). Thus, a critical parameter for realizing high aspect ratio nanoribbons is to operate in a regime in which growth is slow. For example, at low 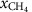 of 0.0066 and high 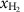 of 0.33, not only is a slow *R*_*l*_ of 40 nm h^−1^ achieved, but also a much slower *R*_*w*_ of 1.4 nm h^−1^, yielding ribbons with an aspect ratio of 30, on average, and as high as 70 (Supplementary Fig. 3). Minimizing *R*_*w*_ also makes it possible to tailor *w* with high precision. Empirical rate laws are identified (insets of Fig. 2a,b), indicating that *R*_*l*_ scales as 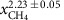 and 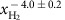. Furthermore, an Arrhenius temperature dependence is observed with an activation energy of 7.2±0.4 eV (Supplementary Fig. 2). For comparison, the activation energy for graphene growth on Cu is only 1–3 eV (ref. 35). However, graphene growth on Cu at atmospheric pressure yields hexagonal crystals instead of nanoribbons^36^, highlighting that different mechanisms control growth.

We also find that the 1D nature of growth is insensitive to the bulk Ge dopant concentration (*N*_Sb_<1.5 × 10^18^ cm^−3^), Ge surface treatment prior to synthesis (OH, H and Cl functionalization), and annealing time before growth (Supplementary Figs 4–6). Nanoribbons are not observed on Ge(110) nor Ge(111) under any growth condition (Supplementary Fig. 7).

### Low-energy electron microscopy and diffraction

**Figure 3:**
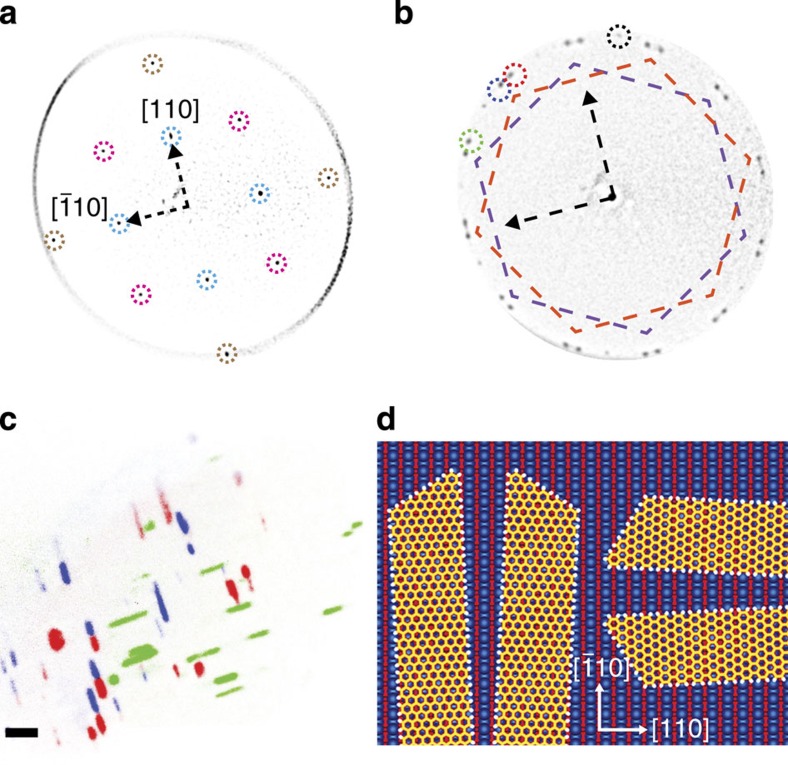
LEEM characterization of graphene nanoribbons on Ge(001). (**a**) Overlaid LEED patterns taken at 135 and 121 eV showing the {01} (cyan), {02} (brown) and {11} (magenta) diffracted beams from the unreconstructed Ge(001) surface. (**b**) LEED pattern obtained at 67 eV showing the purple and orange families of graphene orientations rotated by 30° and the splitting of the spots by 6° within each family. An additional set of sixfold diffraction spots (with one spot circled in black) is also observed, corresponding to a low aspect ratio, non-ribbon crystal with a lattice that is rotated 15° relative to that of the nanoribbons. Arrows indicate the Ge〈110〉 directions as shown in **a**. A fast Fourier transform bandpass filter is applied to the LEED images in **a**,**b** in order to remove the background attributed to diffuse scattering and secondary electrons. (**c**) Superposition of dark-field images taken at 26 eV of graphene nanoribbons on Ge(001) in which green, blue and red channels of the image originate from the {01} graphene diffraction spots circled with the same colour in **b**. Scale bar is 1 μm. The ribbons in **a**–**c** are grown at 910 °C with 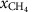 of 0.0092 and 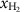 of 0.33 for 6 h. (**d**) Schematic depicting the four nanoribbon orientations that are detected on an unreconstructed Ge(001) surface. The ribbon edges are aligned along the armchair direction of graphene, which is rotated 3° from the Ge〈110〉 directions. The top two layers of Ge atoms are blue while all other layers of Ge atoms are red. It is not yet clear if the ribbons run parallel, perpendicular, or both parallel and perpendicular to the blue rows of Ge atoms.

Low-energy electron microscopy (LEEM) and diffraction (LEED) are used to determine the orientation of the graphene lattice with respect to the underlying Ge and with respect to the ribbon edges. The overlaid LEED patterns in Fig. 3a taken at 121 and 135 eV establish the Ge[110] and 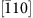 directions. The LEED pattern in Fig. 3b obtained at 67 eV shows that the graphene lattice primarily exists within two families of crystallographic orientations, denoted by the orange and purple hexagons. Dark-field imaging in Fig. 3c indicates that the purple family of diffraction spots originates from ribbons that are oriented with their long axis approximately parallel to Ge[110] whereas the orange family of spots originates from ribbons that are oriented with their long axis approximately parallel to Ge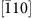. Both of these families of ribbons have edges that are macroscopically aligned with the armchair direction of graphene. This armchair edge orientation is unique because graphene crystals grown on Cu and Ni typically have edges that are aligned along the zigzag direction of graphene^25^,^37^.

Within each family, there are two unique graphene orientations that are rotated 2.9±0.4° (∼3°) relative to the Ge〈110〉 directions, as indicated by the diffraction spots belonging to the orange family that are circled in red and blue in Fig. 3b. These nanoribbon orientations are depicted in Fig. 3d. Dark-field imaging in Fig. 3c indicates that some of the ribbons are single crystalline, corresponding to either the +3° or −3° graphene orientation, whereas others are bi-crystalline, in which the crystal lattice of one half of the ribbon is rotated by 2 × 3°=6° with respect to the other half. This indicates that the nanoribbons nucleate in their centre and then grow in opposite directions along their length. These dynamics potentially explain why the ribbons are elevated in their centre (Fig. 1c); the sublimation of Ge may be locally suppressed under the ribbons as they grow. Interestingly, the lattice of the non-ribbon graphene crystals with lower aspect ratio and edges not aligned on Ge〈110〉, like the one observed in Fig. 1a, is rotated with respect to the lattice of the nanoribbons, typically by 15° as observed in Fig. 3b. This difference indicates that the anisotropic nanoribbon growth is driven only when there is a specific relative orientation between the graphene lattice and the Ge(001) surface.

### Scanning tunneling microscopy

**Figure 4:**
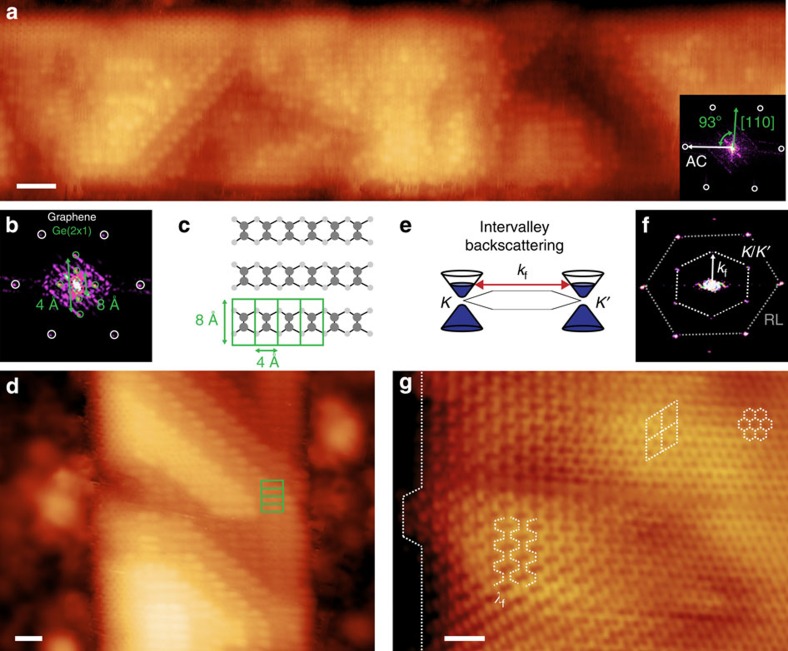
STM studies of graphene nanoribbons on Ge(001). (**a**) STM image of a graphene nanoribbon with *w* of 8.2 nm (scale bar 2 nm, applied bias −1 V, current 400 pA). Inset contains the corresponding FFT illustrating the graphene lattice (white circles) with the armchair (AC) direction forming a 93° angle with the Ge[110] direction. (**b**) High-resolution FFT of the nanoribbon in **a** illustrating the sixfold graphene reciprocal lattice (white circles) in addition to the twofold Ge(001) (2 × 1) dimer lattice with periodicity of 4/8 Å (green circles). (**c**) Schematic illustrating the Ge dimerization and the resulting periodicity. (**d**) STM image of a nanoribbon in which the underlying Ge dimer reconstruction is clearly visible (scale bar 1 nm, applied bias −1 V, current 400 pA). Green boxes correspond to the structure shown in **c**. (**e**) Schematic of the intervalley backscattering process leading to quasiparticle interference patterns in which *k*_f_ is the Fermi wavevector. (**f**) FFT showing the graphene reciprocal lattice (RL) points (outer hexagon) in addition to the six *K*/*K*′ points (inner hexagon) observed due to intervalley backscattering. (**g**) STM image of a nanoribbon in which quantum interference patterns near the edge (armchair-like and rhomboid regions) and regular graphene lattice in the interior (hexagonal region) are observed (scale bar 1 nm, applied bias 25 mV, current 50 pA). The ribbons in **a**,**b**,**d**,**f**,**g** are grown at 910 °C with 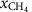 of 0.0092 and 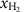 of 0.33 for 1.5 h.

Ultra-high vacuum STM is performed to substantiate the LEED data and to determine the atomic nature of the graphene nanoribbon edges on Ge(001). The STM image and its corresponding fast Fourier transform (FFT) in Fig. 4a indicate that the ribbon edges are straight and parallel to the armchair direction of graphene with little edge roughness and that the graphene lattice is rotated 3° from the Ge〈110〉 directions, consistent with the LEED data. The Ge underneath the nanoribbons retains the common (2 × 1) dimer reconstruction (Fig. 4b–d) even after ambient exposure. Contamination of the bare Ge surface upon exposure to ambient often precludes precise topographical imaging of the nanoribbon edge structure. Using the topographic data alone, we can set an upper limit on the edge roughness. For example, the representative 40 nm ribbon segment in Fig. 4a has roughness of <0.5 nm (two lattice constants of graphene). However, we can learn more about the edge structure from quantum interference patterns caused by intervalley backscattering (Fig. 4e) of charge carriers at the ribbon edges. The ring-like shapes with a 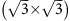
R30° unit cell highlighted with the rhombuses in Fig. 4g and the armchair-like patterns with periodicity (*λ*_f_) of 3.7 Å in Fig. 4g are consistent with electron backscattering at armchair edges^16^,^38^ and are clearly revealed by the prominence of the *K*/*K*′ points in the FFT in Fig. 4f. The presence of these coherent interference patterns combined with the small line edge roughness indicates that the edges consist primarily of smooth armchair segments. The interference patterns decay into the interior of the ribbons with length scales comparable to graphene on SiC^38^ and metals^39^. The atomic structure of the hexagonal graphene lattice is observed past these decay lengths in the interior of the nanoribbons (Fig. 4g).

Scanning tunneling spectroscopy (STS) is used to probe the electronic density of states of nine nanoribbons with *w* ranging from 5 to 19 nm (Supplementary Figs 8–9 and Supplementary Discussion). While it is difficult to make conclusive deductions about the bandgaps from the tunneling spectra due to thermal broadening and tunneling into Ge surface states, the spectra are consistent with those previously reported for semiconducting graphene nanostructures^16^,^17^,^40^,^41^, with suppressed density of states near the Fermi level compared with continuous monolayer graphene on Ge and with the possible development of band edges.

### Charge transport measurements

**Figure 5:**
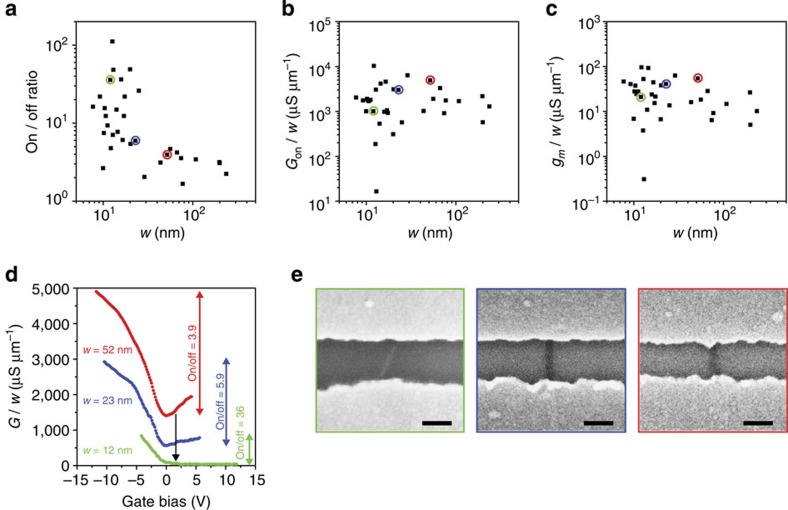
Charge transport of graphene nanoribbon field-effect transistors. (**a**–**c**) On/off current ratio (**a**), on-state conductance per width (*G*_on_/*w*) (**b**) and transconductance per width (g_m_/*w*) (**c**) plotted against *w*. (**d**) Conductance per width (*G*/*w*) plotted against applied back-gate voltage for representative ribbons with *w* of 52 nm (red), 23 nm (blue) and 12 nm (green), respectively. The transfer curves in **d** correspond to the data points circled with the same colour in **a**–**c**. (**e**) Scanning electron micrographs of the nanoribbon channels corresponding to the data points in **a**–**c** and transfer curves in **d** with the same colour. Scale bars in **e** are 100 nm. All measurements are conducted at room temperature in ambient conditions using a source voltage of −0.1 V.

Charge transport measurements are conducted to investigate the electrical properties of 33 nanoribbons using a field-effect transistor geometry (Fig. 5). The ribbons are relatively wide (*w*>10 nm), because these ribbons are more conducive to transfer onto insulating SiO_2_/Si wafers (Fig. 5e). Transfer is required to measure the electrical properties of the ribbons independent of parallel conduction pathways through the Ge substrates used for growth.

The magnitude of the ribbon conductance modulation caused by varying an applied back-gate voltage (that is, the on/off ratio) generally increases as *w* is reduced below 15 nm (Fig. 5a,d), consistent with the opening of a bandgap that scales inversely with *w*. The on/off ratios are comparable to those of high-quality chemically exfoliated nanoribbons of similar *w* (for example, on/off of ∼1, ∼5 and ∼100 have been demonstrated for *w* of ∼50, ∼20 and ∼10 nm, respectively)^20^. However, the largest gains in on/off ratio are not expected until *w* is reduced to 3–5 nm.

The on-state conductance (Fig. 5b) and transconductance (Fig. 5c), normalized by *w*, are not correlated with *w* and, thus, do not generally deteriorate as *w* is decreased. The nanoribbon channel resistance and the Pd–nanoribbon contact resistance are not separately quantified. However, the on-state conductance is high (generally ranging from 500 to 5,000 μS μm^−1^) and the on-state resistance (average of ∼700 Ω μm) is similar to literature values for Pd–graphene contact resistance (150–1,640 Ω μm)^42^,^43^,^44^,^45^,^46^,^47^,^48^,^49^. Therefore, the data suggest that at the relatively short channel length used here of <220 nm, charge transport is not limited by edge scattering or by channel resistance, but instead by the Pd–nanoribbon contacts. The variability in the on-state conductance (Fig. 5b) and the transconductance (Fig. 5c) data is likely caused by contact-length variability, which increases as the ribbons become narrower and thus shorter (Supplementary Discussion).

### Characterization of the graphene/germanium interface

As growth progresses, the ribbons eventually merge to form a continuous graphene film that is self-limiting to a monolayer. We analyse these continuous films to characterize the graphene/Ge interface and the Ge nanofaceting beneath graphene. The films have negligible Raman D-band intensity (Supplementary Fig. 10), indicating an sp^2^ graphene lattice with low defect density and a relatively weak graphene/Ge interaction. Similar to graphene on Ge(110) and Ge(111)^33^, the continuous films can be peeled off of the Ge(001) surface using a thin Au layer, indicating that the interaction strength is <60 meV (ref. 50). This weak interaction is consistent with the STM data, which show that the underlying Ge(2 × 1) reconstruction is unaffected by the ribbons (Fig. 4b–d) and that the quantum interference patterns near the ribbon edges are not disrupted by the substrate (Fig. 4f,g).

Raman spectroscopy shows that the graphene films on Ge(001) are stable in ambient conditions, even at 200 °C for >24 h, and X-ray photoelectron spectroscopy (XPS) indicates that the underlying Ge(001) remains unoxidized in ambient conditions for >4 weeks after growth (Supplementary Fig. 10). Thus, the direct synthesis of nanoribbons on Ge(001) yields a pristine nanoribbon/substrate interface compared to that of ribbons deposited from solution or transferred from another surface, during which disorder and impurities are introduced.

### Germanium nanofacet formation

**Figure 6:**
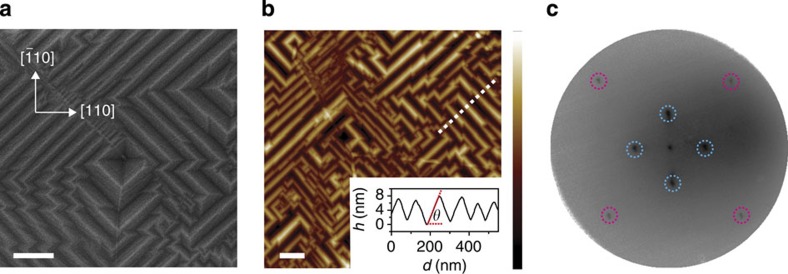
Characterization of continuous graphene films grown on Ge(001). (**a**,**b**) SEM image (**a**) and AFM topographic map (**b**) of a graphene monolayer on Ge(001). Scale bars, 200 nm (**a**,**b**); height scale bar, 11.8 nm (**b**). Inset of **b** is height (*h*) plotted against projected surface distance (*d*) showing the profile of the facets across the dotted line in **b**. (**c**) LEED pattern taken at 19 eV showing the {01} spots from the unreconstructed Ge(001) surface (magenta) and the {00} spots from the Ge facets (cyan).

As previously observed in Figs 1b,c and 4a, the Ge surface selectively forms nanofacets underneath the nanoribbons. These hill-and-valley structures are similar to surface faceting that has been observed for other combinations of surfaces and adsorbates^51^. These facets are more easily visualized and characterized below continuous graphene films (Fig. 6a). The facet angle (Fig. 6b inset) is shallow for thin ribbons and becomes steeper as the ribbons grow wider and eventually merge to form a continuous graphene film. Underneath continuous films, the angle measured via AFM (Fig. 6b), LEED (Fig. 6c) and X-ray reflectivity (XRR; Supplementary Methods) is 8.1±1.1°, 8.5±1.0° and 7.8±1.1°, respectively, which is consistent with the Ge(107) facet. Interestingly, the faceting below the nanoribbons is reversible upon annealing at 800 °C in high vacuum, resulting in a planar interface (Supplementary Fig. 11).

## Discussion

Our experimental data, which show that growth is anisotropic only when the armchair direction of graphene is rotated 3° from the Ge〈110〉 directions, provide insight into the mechanisms governing the 1D nature of growth. It is likely that this crystallographic relationship is set during the early stages of nucleation; as the graphene nucleus becomes larger, the energy barrier associated with its rotation significantly increases, which, consequently, fixes the orientation of the nanoribbon lattice during the subsequent growth^52^. The Ge(001) surface consists of two types of terraces that have the same structure but are rotated 90° with respect to each other. It is plausible that on one set of terraces, the armchair direction of the graphene nuclei is rotated 3° from Ge[110] and on the other set of terraces, the armchair direction is rotated 3° from Ge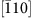, giving rise to the two families of orientations observed in the LEED and STM data. Nucleation does not seem to be strongly correlated with the density or directionality of steps that exist before nucleation (Supplementary Figs 12–14 and Supplementary Discussion).

After nucleation, several factors could drive anisotropic ribbon evolution. While step edges may play a role in setting the crystallographic orientation of the nuclei on each terrace, subsequent preferential growth along step edges cannot solely account for the formation of ribbons with smooth edges over long segments. Furthermore, we do not observe residual nanoparticles at the ribbon ends or tapered ribbon growth that might indicate that the ribbon evolution is driven by a catalyst particle like in conventional nanowire growth^53^. Anisotropic diffusion of intermediate hydrocarbons on the Ge surface cannot alone account for the ribbon evolution because low aspect ratio crystals are also observed.

We hypothesize that the anisotropic growth is due to preferential attachment of intermediate hydrocarbons from the Ge surface to the short (faster growing) ribbon edges over the long (slower growing) ribbon edges, which is consistent with the observation that the low aspect ratio crystals have different lattice orientation than that of the nanoribbons. During attachment, the transition state and the corresponding energy barrier will depend on the relative orientation between the nanoribbon edge and the Ge surface. Similar growth phenomena, such as the formation of polygonal graphene crystals with relatively straight edges and sharp vertices^54^ and the synthesis of other 1D structures on the (001) face of zinc-blende semiconductors^55^,^56^,^57^, have also been attributed to attachment-limited kinetics. The increase in *R*_*w*_ with time (Fig. 1d) may indicate that the barrier for hydrocarbon attachment to the long (slower growing) ribbon edges decreases as *w* increases. *R*_*w*_ begins to noticeably increase after *w* exceeds about 30 nm, potentially corresponding to when ribbons begin to outgrow the Ge terrace on which they nucleate. The Ge hill-and-valley structures increase in height and width as *w* increases; this evolution may also perturb the interactions of the nanoribbon edges with the underlying Ge and, thus, may also modify the attachment barrier.

Several challenges in synthesis and fabrication have inhibited progress in nanoribbon research and development. Specifically, (1) the synthesis of nanoribbons with armchair edges, (2) the definition of nanoribbons with width <10 nm and (3) the direct integration of nanoribbons onto insulating or semiconducting platforms have been difficult. This work overcomes these challenges. We demonstrate that by controlling graphene synthesis on Ge(001) via CVD, it is possible to realize oriented nanoribbons with sub-10 nm width, controlled crystallographic orientation and smooth armchair edges. This direct, self-defining growth offers precise control over the ribbon structure beyond the fidelity of top-down lithography and yields a relatively pristine nanoribbon/substrate interface. The ribbons are self-aligning, and ribbons with *w*<10 nm can still be hundreds of nanometres in length, opening the door for exploration of dense nanoribbon arrays as logic, photonic and sensing components in integrated circuits. Improved control over the ribbon placement will enhance the viability of these applications. These results are also technologically important because they enable a scalable, high throughput pathway for integrating nanoribbons directly on conventional large-area semiconductor wafer platforms that are compatible with planar processing, like Ge wafers and potentially epitaxial Ge films on Si (ref. 33) or GaAs (ref. 58) wafers.

## Methods

### Graphene synthesis

Before growth, Ge(001) (Wafer World, resistivity >40 Ω cm, miscut <1°), Ge(110) (University Wafer, resistivity 0.1–0.5 Ω cm with Ga or Sb dopants) and Ge(111) (Semiconductor Wafer, resistivity >30 Ω cm) substrates are cleaned via sonication in acetone and isopropyl alcohol for 15 min followed by etching in deionized H_2_O (18 MΩ cm) at 90 °C for 15 min. The Ge substrates are loaded into a horizontal tube furnace with a quartz tube inner diameter of 34 mm and the system is evacuated to ∼10^−6^ torr. The system is then filled to atmospheric pressure with a mixture of Ar (99.999%) and H_2_ (99.999%) using a constant total flow rate of 300 s.c.c.m. The Ge samples are annealed for 30 min at the selected growth temperature and then CH_4_ (99.99%) is introduced to begin the synthesis. In order to terminate growth, samples are rapidly cooled in the same atmosphere used during synthesis by sliding the furnace away from the growth region. Specific growth conditions are provided in Supplementary Table 1.

### Graphene transfer

In order to transfer the ribbons from Ge to SiO_2_/Si for Raman spectroscopy and charge transport measurements, poly(methyl methacrylate) (PMMA) is spin coated onto the graphene/Ge substrate. The PMMA/graphene/Ge stack is floated on a solution of 28:1:1 H_2_O:HF:H_2_O_2_ to etch the Ge layer. The PMMA/graphene stack is transferred to H_2_O to clean the ribbons and then lifted out of the H_2_O using a SiO_2_/Si substrate. The PMMA is dissolved in acetone and the sample is washed with isopropyl alcohol.

### Characterization

After growth, the samples are characterized with SEM (Zeiss LEO 1530) and AFM (Veeco MultiMode SPM) in tapping mode. For each nanoribbon synthesis, the *w* and *l* of ∼250 ribbons are measured from SEM images using ImageJ. Raman spectroscopy (Thermo Scientific DXRxi) in Supplementary Fig. 1 is performed using excitation wavelength of 532 nm, power of 10 mW and spot size of 0.6 μm. Graphene degradation studies in Supplementary Fig. 10 are conducted with Raman spectroscopy (Horiba Jobin Yvon LabRAM Aramis) with excitation wavelength of 442 nm, power of 0.01 mW and spot size of 1 μm. XPS (Thermo Scientific K-Alpha) is performed with spot size of 30 μm and energy resolution of 0.57 eV. LEEM and LEED (SPECS Fe-LEEM/PEEM P90) are conducted using incident electron energies of ∼25 eV and between 19–135 eV, respectively. STM and STS (Omicron VT, base pressure of 10^−12^ mbar) are performed simultaneously at 300 K using electrochemically etched W tips. STS data is generated by superimposing a 30 mV modulation at 10 kHz on top of the bias voltage and analysing the tunnelling current with a SR830 Lock-In Amplifier. XRR measurements (Bruker D8 Discover with a VÅNTEC 500 Area Detector) are performed with a Cu Kα X-ray source.

### Device fabrication

The nanoribbons are transferred onto SiO_2_/Si wafers as described, above. Electron-beam lithography is used to define source and drain electrodes with channel lengths between 75 and 220 nm. Thermal evaporation is used to deposit Pd electrodes 35 nm in thickness. The back-gate electrode and back-gate dielectric are the Si wafer and 15 nm of thermally grown SiO_2_, respectively. Each field-effect transistor is measured at room temperature in ambient conditions using a source voltage of −0.1 V.

## Additional information

**How to cite this article:** Jacobberger, R. M. *et al.* Direct oriented growth of armchair graphene nanoribbons on germanium. *Nat. Commun.* 6:8006 doi: 10.1038/ncomms9006 (2015).

## Supplementary Material

Supplementary Information Supplementary Figures 1-14, Supplementary Table 1, Supplementary Discussion, Supplementary Methods and Supplementary References
