# Supplementary material for: Direct oriented growth of armchair graphene nanoribbons on germanium
Source: Nat Commun. 2015 Aug 10;6:8006. doi: 10.1038/ncomms9006 (PMC4918381; doi:10.1038/ncomms9006)
Supplement: Supplementary Information — Supplementary Figures 1-14, Supplementary Table 1, Supplementary Discussion, Supplementary Methods and Supplementary References [file ncomms9006-s1.pdf]

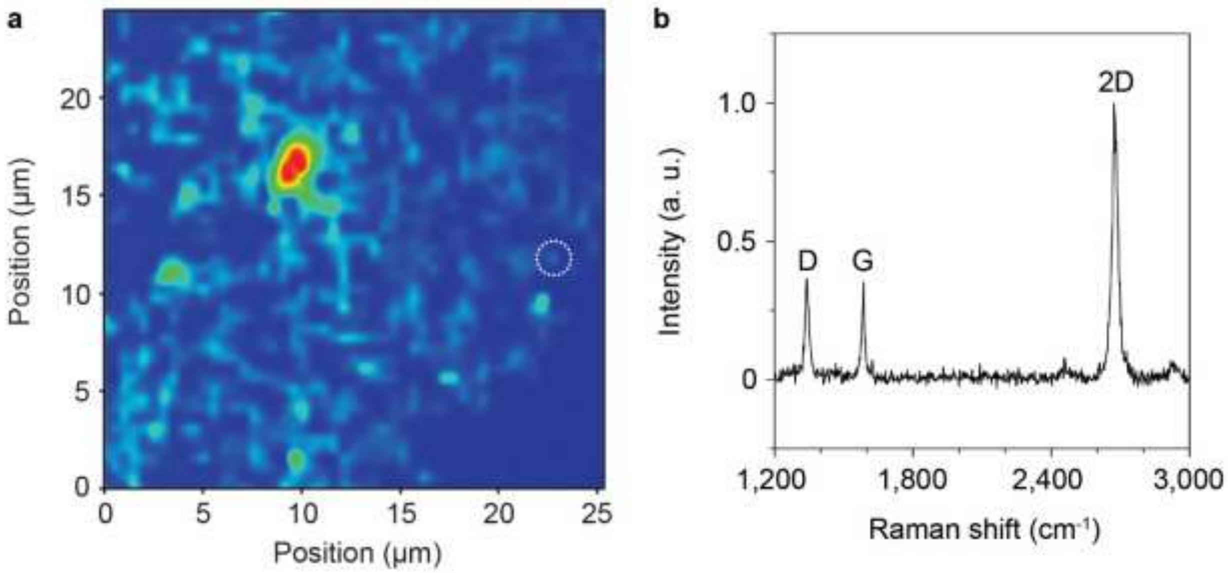

Supplementary Figure 1 Raman imaging of graphene nanoribbons transferred onto SiO_2/Si. Raman spectroscopy confirms that the nanoribbons are graphene. In order to improve the signal-to-noise ratio of the spectra, ribbons with large average *w* of 74 nm are transferred to SiO_2/Si substrates. **a,** Raman scattering from individual ribbons is measured via spectral mapping. The Raman scattering intensity at 1340 cm^{-1}, corresponding to the D peak of graphene, is plotted against position on the sample surface in **a**. **b,** Representative Raman spectrum from a ribbon with *w* of 35 nm, which is located in the dashed circle in **a**. *w* is determined from imaging this same area with SEM following Raman imaging. Raman scattering is observed at 1,587 cm^{-1} and 2,677 cm^{-1}, corresponding to the G and 2D bands of graphene, respectively. The ratio of the areas of the 2D and G peaks and the 2D full-width-at-half-maximum are 6.0 and 28 cm^{-1}, respectively, indicating that the ribbons are single-layer graphene[1]. The D peak at 1,341 cm^{-1} could, in part, be due to the presence of defects or adsorbates, which are likely introduced to some extent during the graphene transfer. However, the D peak is also expected to be strongly active at pristine armchair edges[2]. The ribbons are synthesized at 910 °C using x_{CH4} of 0.0092 and x_{H2} of 0.33 for 6 h.

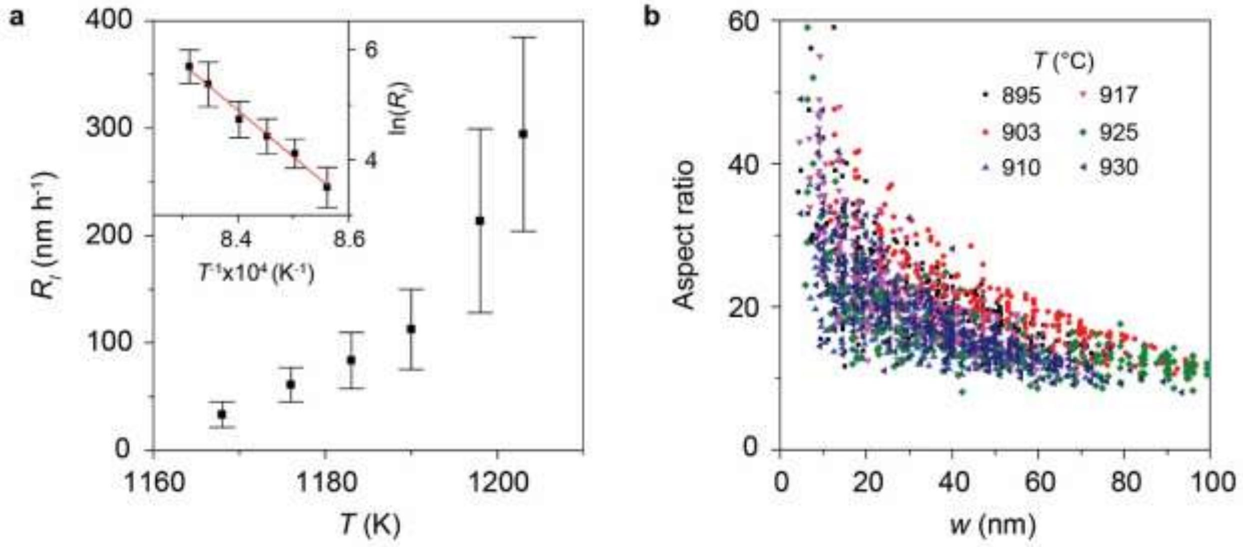

Supplementary Figure 2 Effect of growth temperature on graphene nanoribbon growth on Ge(001). **a,** R_l plotted against T. Inset of **a** contains the Arrhenius plot of ln(R_l) vs. T^{-1} with the best fit line in red, which is used to calculate the activation energy for growth. Error bars indicate standard deviation. **b,** Aspect ratio plotted against w for growths with varying T between 895 and 930 °C. Unlike with varying x_{CH4} and x_{H2}, we do not observe an obvious effect of T on the aspect ratio. The ribbons in **a,b** are synthesized with constant x_{CH4} of 0.0092 and x_{H2} of 0.33 and varying T of 895 °C and t of 10 h (black squares in **b**), T of 903 °C and t of 7.25 h (red circles in **b**), T of 910 °C and t of 3 h (blue upward triangles in **b**), T of 917 °C and t of 2.5 h (purple downward triangles in **b**), T of 925 °C and t of 2 h (green diamonds in **b**), and T of 930 °C and t of 1 h (navy leftward triangles in **b**).

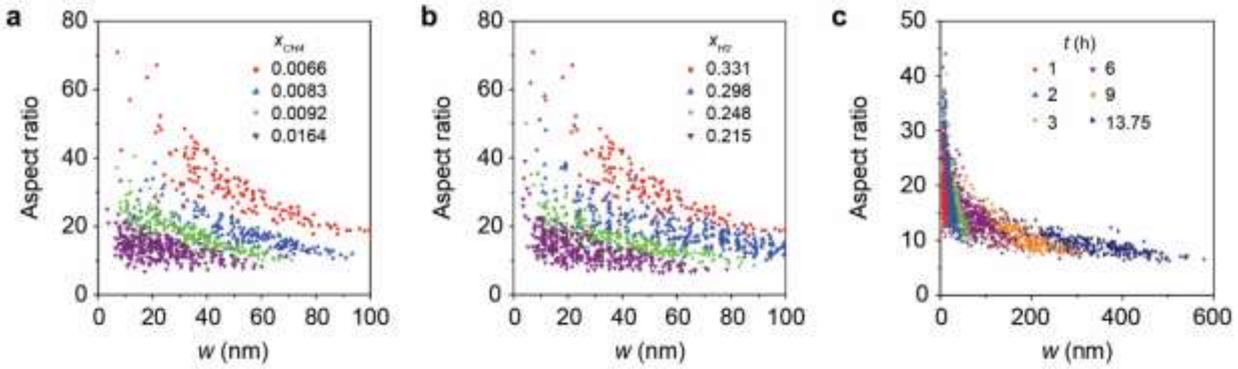

Supplementary Figure 3 Effect of precursor composition on graphene nanoribbon growth on Ge(001). a-c, Ribbon aspect ratio plotted against w for growths with varying x_{CH4} (**a**), x_{H2} (**b**), and t (**c**). Ribbons in **a** are grown at 910 °C with x_{H2} of 0.33 and varying x_{CH4} of 0.0066 and t of 18.25 h (red squares), x_{CH4} of 0.0083 and t of 6 h (blue upward triangles), x_{CH4} of 0.0092 and t of 3 h (green circles), and x_{CH4} of 0.0164 and t of 0.5 h (purple downward triangles). Ribbons in **b** are grown at 910 °C and x_{CH4} of 0.0066 with varying x_{H2} of 0.331 and t of 18.25 h (red squares), x_{H2} of 0.298 and t of 8.5 h (blue upward triangles), x_{H2} of 0.248 and t of 2 h (green circles), and x_{H2} of 0.215 and t of 0.75 h (purple downward triangles). Ribbons in **c** are grown at 910 °C, x_{H2} of 0.33, and x_{CH4} of 0.0092 with varying t of 1 h (red squares), 2 h (blue upward triangles), 3 h (green circles), 6 h (purple downward triangles), 9h (orange leftward triangles), and 13.75 h (navy rightward triangles). The data in **c** show that for constant x_{CH4} and x_{H2}, the aspect ratio decreases with t, which is presented in a different form in Fig. 1d-f in the main text. However, for a given w, ribbons grown at lower x_{CH4} and higher x_{H2} have higher aspect ratio, as shown in **a,b**. The decreasing aspect ratio with increasing w in **a,b** can be explained by the trend in **c** and the polydispersity in w and l. Ribbon nucleation likely occurs continuously throughout growth. Thus, ribbons that nucleate immediately after the synthesis begins have larger w and lower aspect ratio than ribbons that nucleate near the end of the synthesis.

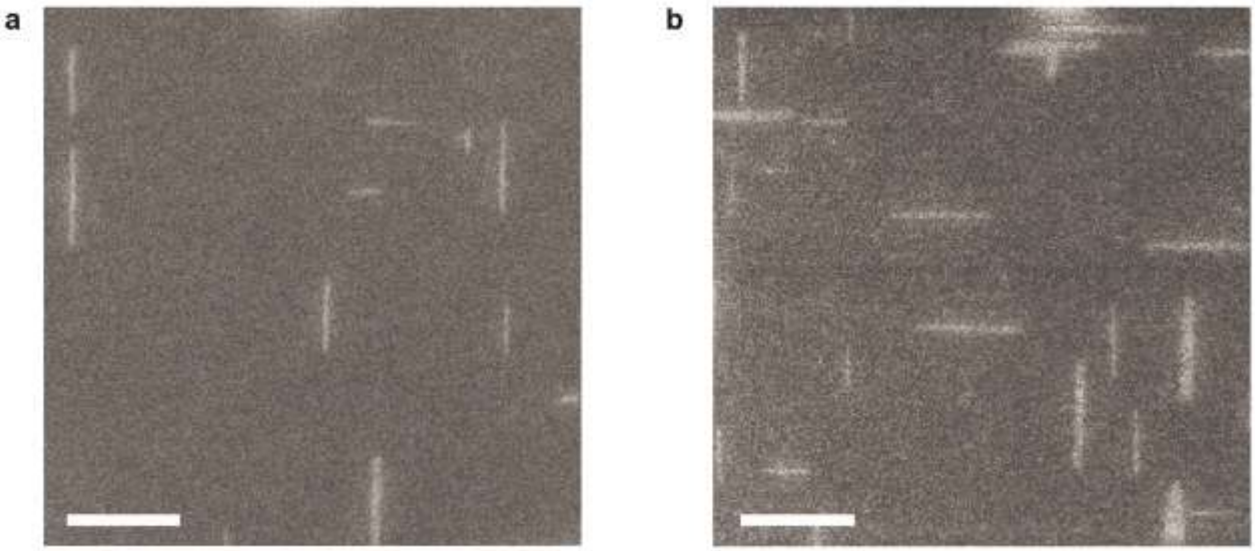

Supplementary Figure 4 Effect of Ge(001) doping concentration on graphene nanoribbon growth. a,b, Scanning electron micrograph of nanoribbons grown on Ge(001) with no extrinsic doping (**a**) and Sb dopant concentration of $1.5x10^{18}$ cm^{-3} (**b**). The anisotropic ribbon evolution is independent of the doping concentration. Ribbons in **a,b** are grown at 910 °C with x_{CH4} of 0.0092 and x_{H2} of 0.33 for 1.5 h. Scale bars are 400 nm.

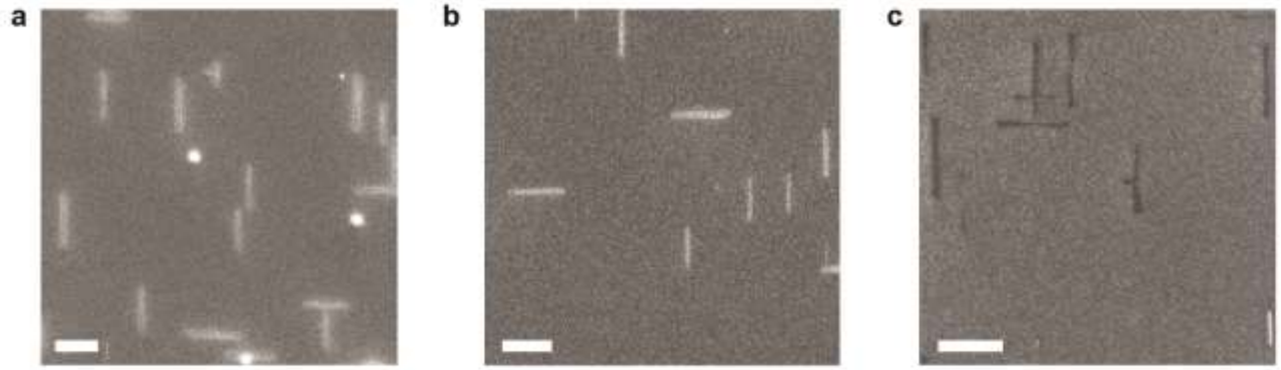

Supplementary Figure 5 Effect of Ge(001) surface treatment on graphene nanoribbon growth. a-c, Ge(001) wafers are terminated with OH (**a**), Cl (**b**), and H (**c**) groups before growth by submerging the wafers in concentrated H_2O, HCl (36.5 % to 38 % by weight), and HF (49 % by weight), respectively. The one-dimensional nature of growth is independent of the surface termination prior to growth. This suggests that these functional groups desorb from the Ge(001) surface at the high temperatures used for growth. The ribbons are grown at 910 °C with x_{CH4} of 0.0066 and x_{H2} of 0.26 for 3 h. Scale bars are 1 μm.

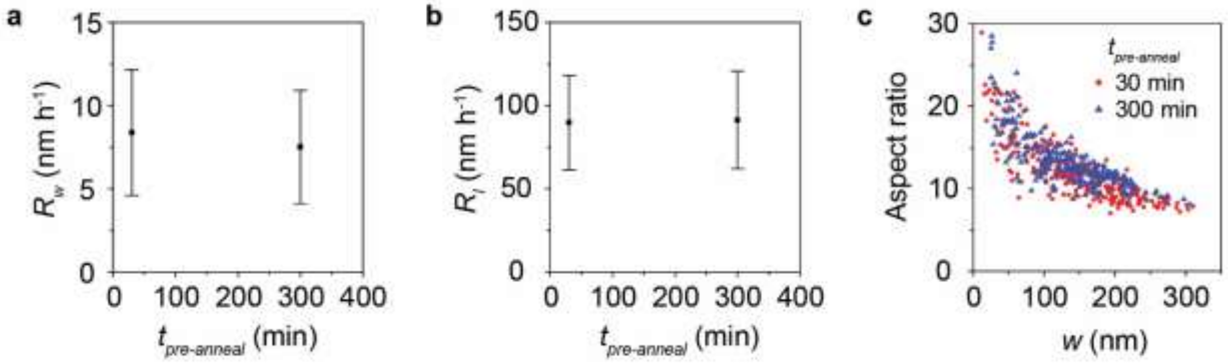

Supplementary Figure 6 Effect of pre-anneal time on graphene nanoribbon growth on Ge(001). a,b, R_w (**a**) and R_l (**b**) plotted against pre-anneal time prior to growth ($t_{pre-anneal}$). Error bars indicate standard deviation. **c,** Ribbon aspect ratio vs. w for $t_{pre-anneal}$ of 30 min (red circles) and 300 min (blue triangles). We find that R_l, R_w, and the aspect ratio are insensitive to $t_{pre-anneal}$. Growths are conducted at 910 °C with x_{CH4} of 0.0092 and x_{H2} of 0.33 for 9 h.

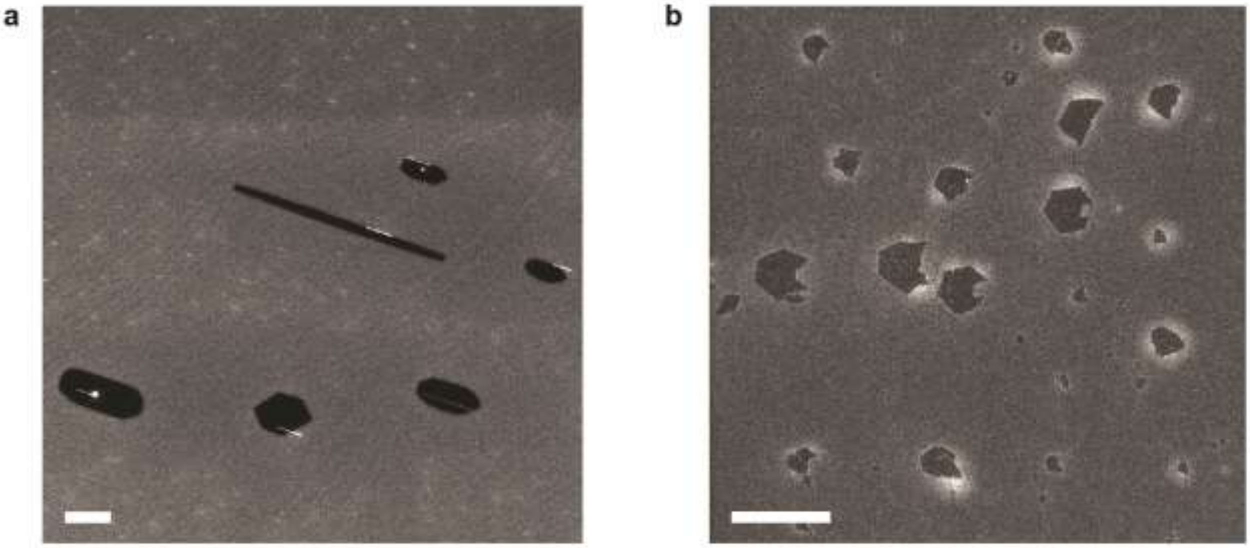

Supplementary Figure 7 Graphene growth on Ge(110) and Ge(111). a,b, Scanning electron micrographs showing representative graphene crystal morphology after growths on Ge(110) (**a**) and Ge(111) (**b**). Similar graphene crystal morphology is observed using most of the conditions listed in Supplementary Table 1. Graphene does not nucleate as nanoribbons on either surface, indicating that the one-dimensional nature of growth is unique to Ge(001). While graphene occasionally nucleates as high-aspect ratio structures on Ge(110), these crystals occur with low yield (5 to 10 %) and their structure and properties are not thoroughly characterized here. The graphene crystals on Ge(110) in **a** are grown at 910 °C with x_{CH4} of 0.0066 and x_{H2} of 0.30 for 8.5 h. The graphene crystals on Ge(111) in **b** are grown at 910 °C with x_{CH4} of 0.0040 and x_{H2} of 0.20 for 2 h. Scale bars are 1 μm.

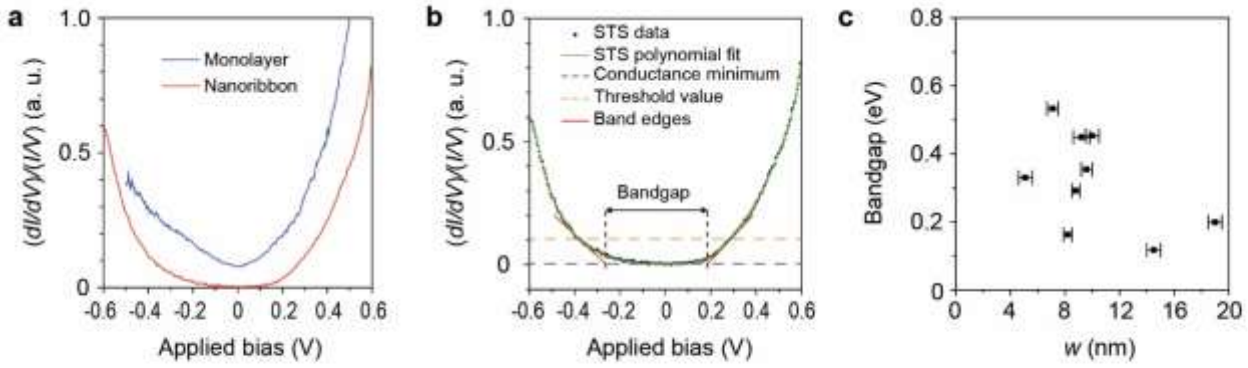

Supplementary Figure 8 Summary of scanning tunneling spectroscopy (STS) of graphene nanoribbons on Ge(001). a, Plot of normalized differential conductance [*dI/dV/(I/V)*] vs. applied bias from a representative graphene nanoribbon with *w* of 10 nm on Ge(001) (red) and a continuous graphene monolayer film on Ge(001) (blue). **b**, Plot of the normalized differential conductance vs. applied bias from the same representative graphene nanoribbon from **a** (black dots), in which the polynomial fit (green line), conductance minimum (blue dashed line), threshold value (orange dashed line), band edges (red lines), and corresponding measured bandgap (450 meV) are shown (see Supplementary Discussion). **c**, Plot of experimentally measured bandgap vs. *w*. The ribbons in **a-c** are grown at 910 °C with x_{CH4} of 0.0092 and x_{H2} of 0.33 for 1.5 h.

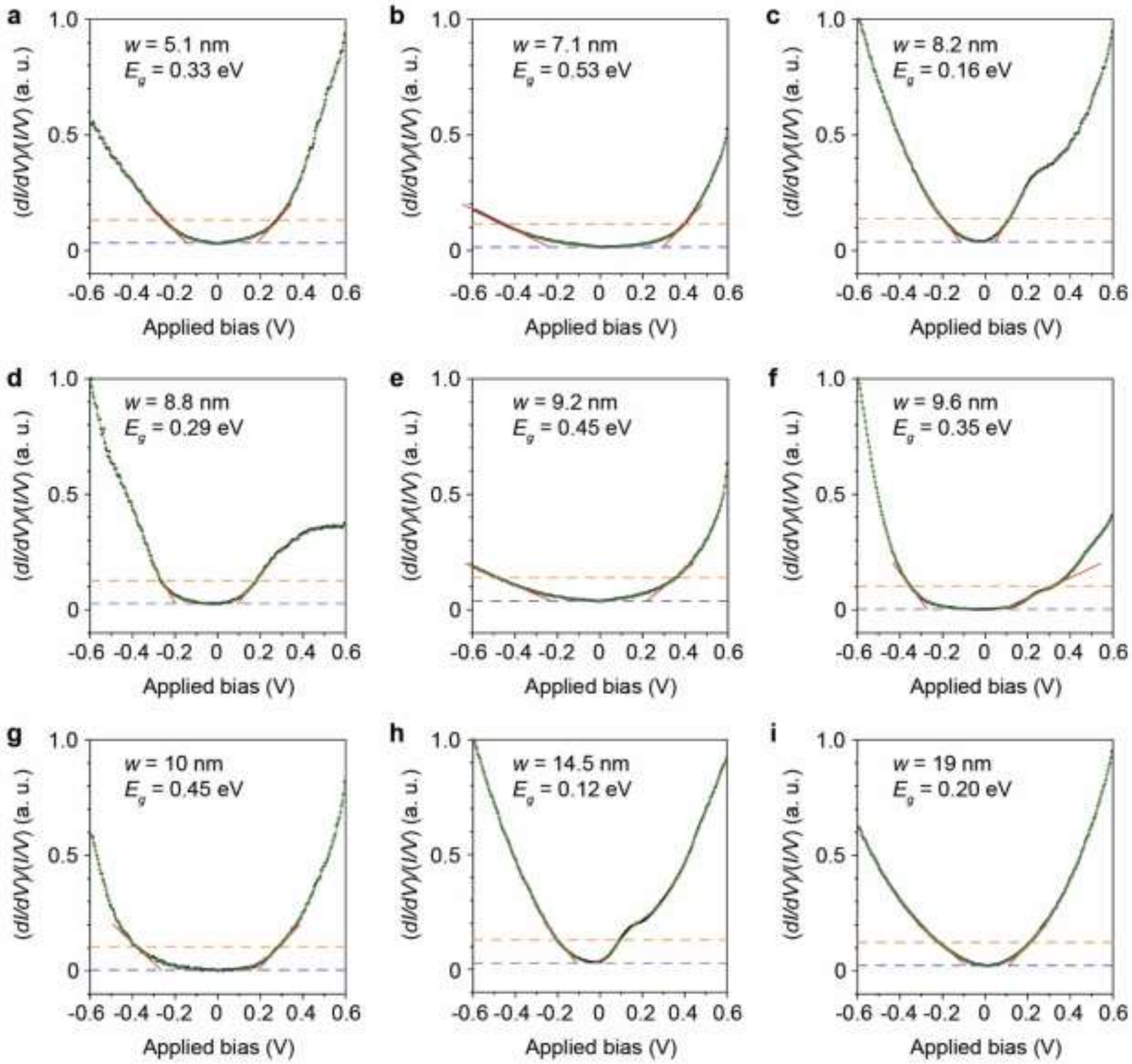

Supplementary Figure 9 Scanning tunneling spectroscopy (STS) of graphene nanoribbons on Ge(001). a-i, Plot of normalized differential conductance [*dI/dV/(I/V)*] vs. applied bias (black dots) from ribbons with width of 5.1 nm (**a**), 7.1 nm (**b**), 8.2 nm (**c**), 8.8 nm (**d**), 9.2 nm (**e**), 9.6 nm (**f**), 10 nm (**g**), 14.5 nm (**h**), and 19 nm (**i**), in which the polynomial fit (green line), conductance minimum (blue dashed line), threshold value (orange dashed line), and band edges (red lines) are shown. The corresponding plot of experimentally measured bandgap vs. *w* is provided in Supplementary Fig. 8c.

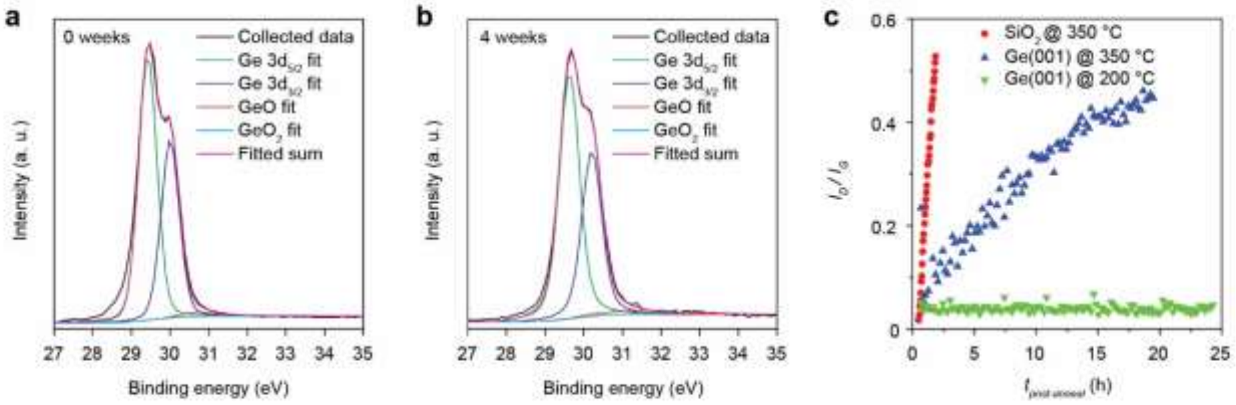

Supplementary Figure 10 Oxidation resistance of the graphene-coated Ge(001) surface and degradation studies of continuous graphene films on Ge(001). a,b, The intensity of the x-ray photoelectron spectroscopy (XPS) signal vs. binding energy (black) after 0 weeks (**a**) and 4 weeks (**b**) of being stored in ambient laboratory conditions. The Ge $3d_{5/2}$ (green), Ge $3d_{3/2}$ (blue), GeO (red), and GeO_2 (cyan) peaks are fit with Voigt curves. The sum of the fitted peaks is given in magenta. Even after 4 weeks, the concentrations of GeO and GeO_2 are below the resolution of the XPS (0.1 %). **c,** The ratio of the areas of the Raman D and G bands (I_D / I_G) plotted against post-annealing time after growth ($t_{post\text{-}anneal}$) in ambient laboratory conditions at 350 °C for graphene transferred to SiO_2 (red circles) and on Ge(001) (blue upward triangles) and at 200 °C for graphene on Ge(001) (green downward triangles). At $t_{post\text{-}anneal}$ of 0 h, the I_D / I_G is negligible, indicating an sp^2 graphene lattice with low defect density. In ambient conditions at 350 °C, graphene on Ge(001) is more stable than graphene transferred to SiO_2, as indicated by the slower increase in I_D / I_G over time. This is important because graphene degradation has been shown to be directly related to the oxidation of the underlying surface[3]. Once the graphene begins to degrade, oxygen can permeate the graphene through the defective sites to oxidize the underlying surface. Furthermore, graphene grown on Ge(001) is stable at 200 °C for > 24 h, which is greater than the temperature at which electronic devices are typically operated.

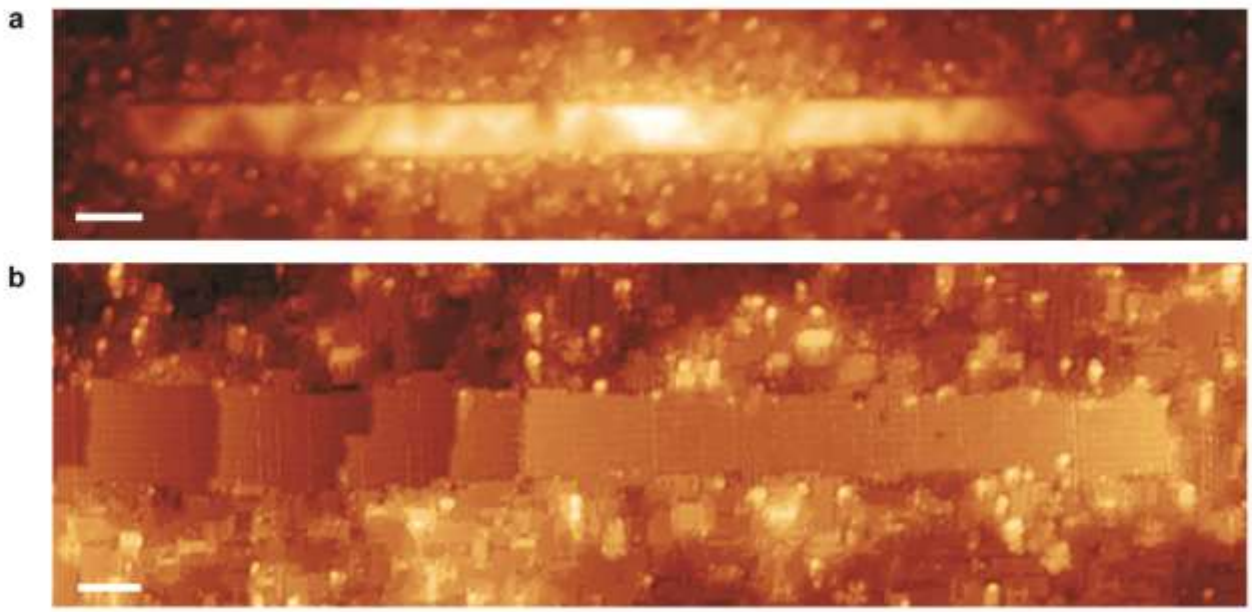

Supplementary Figure 11 Planarization of Ge facets beneath graphene nanoribbons. a,b, Scanning tunneling micrographs of a graphene nanoribbon after growth on Ge(001) (scale bar 10 nm, applied bias -2 V, current 200 pA) (**a**) and a different ribbon on the same substrate after further annealing at 800 °C in ultra-high vacuum (scale bar 10 nm, applied bias -1 V, current 200 pA) (**b**). The height modulation below the ribbon in **a** is due to Ge facet formation. After annealing, the Ge surface underneath the ribbon becomes planar and the underlying rows in the reconstructed (3x1) Ge(001) surface align 3° from the long axis of the ribbon. The nanoribbons in **a,b** are grown at 910 °C using x_{CH4} of 0.0092 and x_{H2} of 0.33 for 1.5 h.

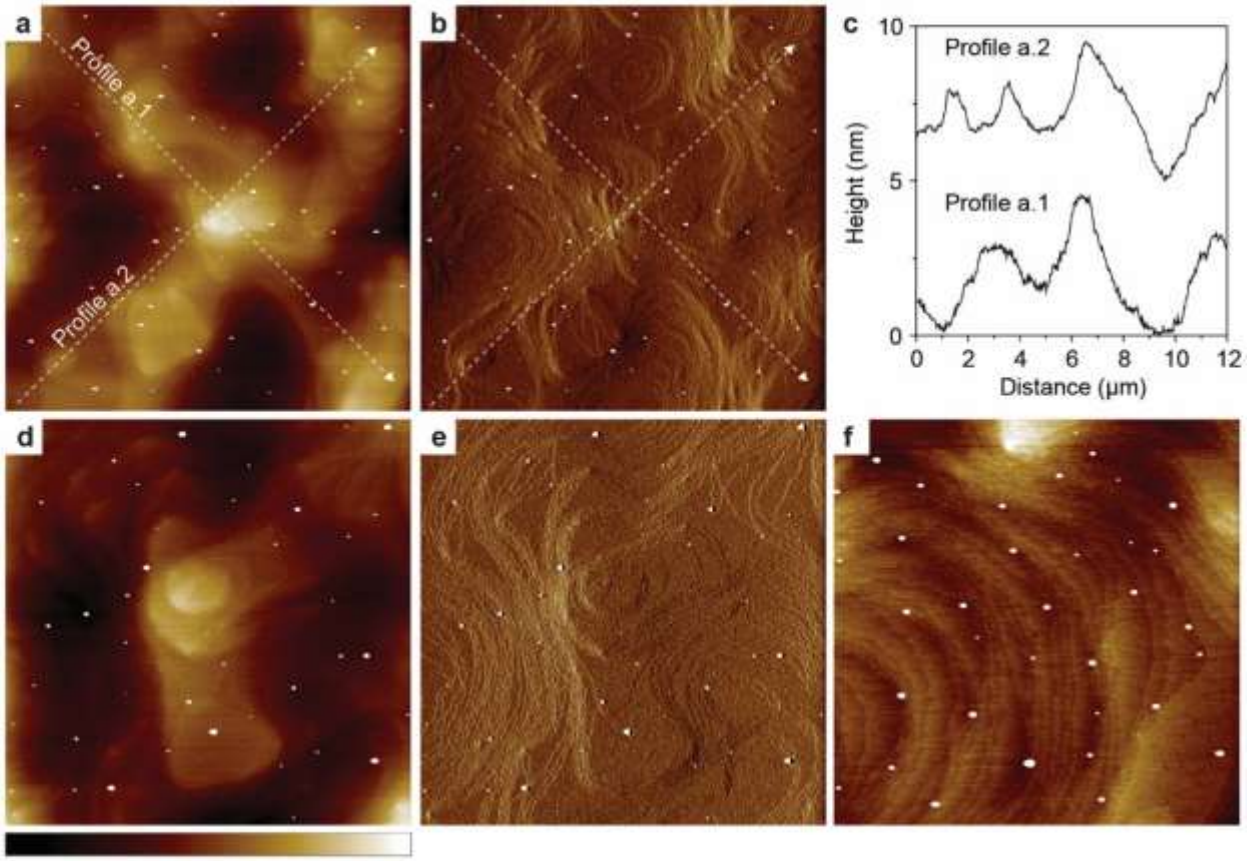

Supplementary Figure 12 Atomic force microscopy (AFM) of annealed Ge(001) surface before nanoribbon growth. The Ge(001) surface in **a-f** is annealed at 910 °C with x_{CH4} of 0 and x_{H2} of 0.33 for 0.5 h. **a,b,** Height image (**a**) and corresponding amplitude error image (**b**) with scan size of 10 μm. Height scale bar in **a** is 10 nm and amplitude error scale bar in **b** is 6 mV. **c,** Height profiles along the corresponding dashed arrows in **a** that show the gradually rolling surface morphology. The origin of each arrow in **a** corresponds to the y-axis in **c**. **d,e,** Height image (**d**) and corresponding amplitude error image (**e**) with scan size of 5 μm. Height scale bar in **d** is 5 nm and amplitude error scale bar in **e** is 5 mV. **f,** Height image with scan size of 3 μm. Height scale bar in **f** is 2 nm. Steps in the Ge surface are more distinguished in the amplitude error images in **b,e** than in their corresponding height images in **a,d.**

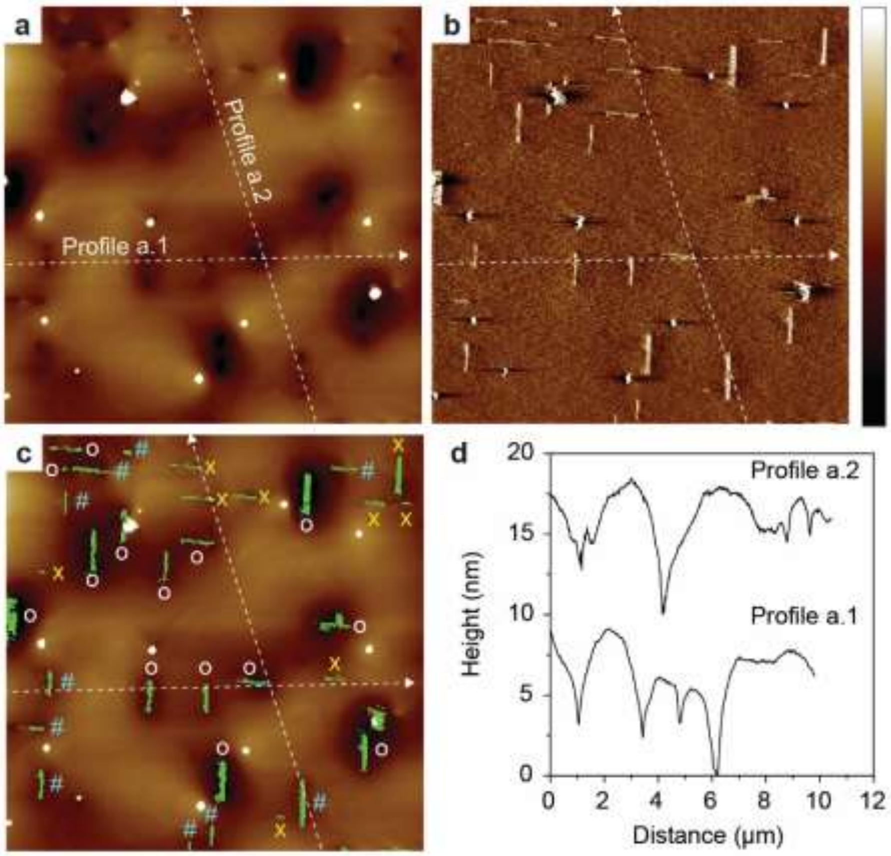

Supplementary Figure 13 Atomic force microscopy (AFM) of graphene nanoribbons on Ge(001). **a,b,** Height image (**a**) and corresponding phase image (**b**) with scan size of 10 μm. Height scale bar in **a** is 20 nm and phase scale bar in **b** is 2°. **c,** Same height image in **a** but with nanoribbons shown in green, as detected from the phase image in **b**, to more clearly show the location of the ribbons with respect to surface features. The white "o", yellow "x", and blue "#" symbols in **c** correspond to ribbons near low points, near high points, and on sloped regions, respectively, of the rolling surface morphology formed during annealing. **d,** Height profiles along the corresponding dashed arrows in **a**. The origin of each arrow in **a** corresponds to the y-axis in **d**. The regions where ribbons are located often correspond to depressions in the Ge(001) surface, which are superimposed on the gradually rolling morphology.

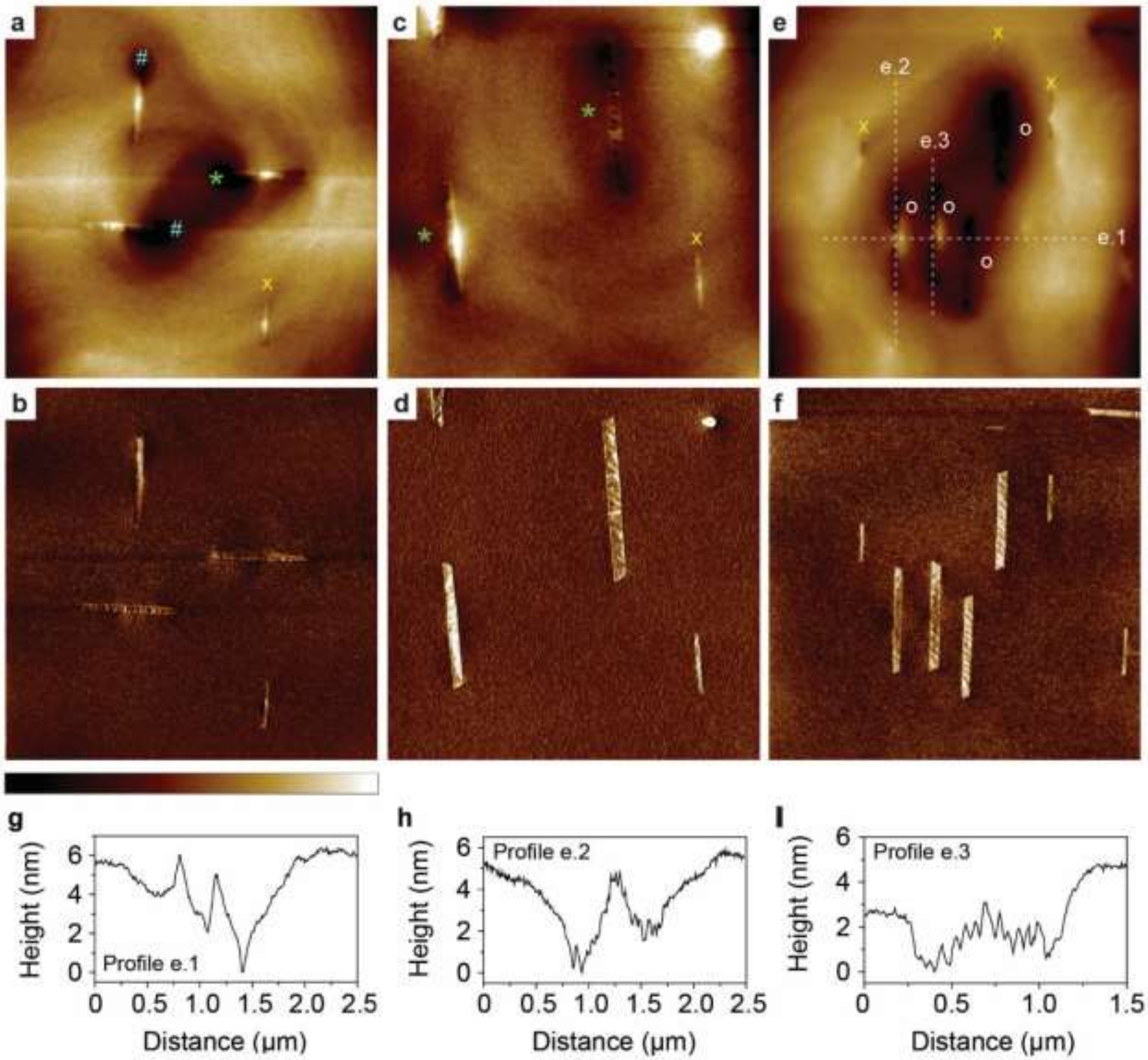

Supplementary Figure 14 Atomic force microscopy (AFM) of graphene nanoribbons on Ge(001). **a,b,** Height image (**a**) and corresponding phase image (**b**) with scan size of 3.3 μm. Height scale bar in **a** is 5 nm and phase scale bar in **b** is 3°. **c,d,** Height image (**c**) and corresponding phase image (**d**) with scan size of 2.5 μm. Height scale bar in **c** is 6 nm and phase scale bar in **d** is 7°. **e,f,** Height image (**e**) and corresponding phase image (**f**) with scan size of 3.3 μm. Height scale bar in **e** is 9 nm and phase scale bar in **f** is 5°. The nanoribbons are more distinguished in the phase images in **b,d,f** than in the corresponding height images in **a,c,e**. The blue "#", green "*", white "o", and yellow "x" symbols correspond to ribbons in which only one end is in a depression, both ends are in depressions, the entire ribbon is in a depression,

and depressions are not observed, respectively. **g-i,** Height profiles along the corresponding dashed lines in **e** that highlight the Ge nanofacets that form underneath the nanoribbons.

Supplementary Table 1 Summary of graphene nanoribbon growth conditions. The table contains the growth conditions used in this work, including the growth temperature (*T*), time (*t*), Ar flux (*Ar*), H_2 flux (H_2), CH_4 flux (CH_4), number of ribbons analyzed (*n*), ribbon width (*w*) and standard deviation (σ_w), ribbon length (*l*) and standard deviation (σ_l), ribbon aspect ratio (*a.r.*) and standard deviation (σ_{ar}), and growth rate of ribbon width (R_w) and length (R_l).

T (°C)	*t* (h)	*Ar* (sccm)	H_2 (sccm)	CH_4 (sccm)	*n*	*w* (nm)	σ_w (nm)	*l* (nm)	σ_l (nm)	*a.r.*	σ_{ar}	R_w (nm h^{-1})	R_l (nm h^{-1})
910	1	200	100	2.8	178	9.79	4.02	182	42	19.0	4.3	4.90	91.0
910	2	200	100	2.8	482	18.5	8.3	341	98	20.2	5.5	4.63	85.3
910	3	200	100	2.8	260	30.9	14.9	501	158	18.0	5.0	5.15	83.5
910	6	200	100	2.8	290	74.0	36.4	986	364	14.5	3.6	6.17	82.2
910	9	200	100	2.8	279	151	68	1617	512	12.0	3.6	8.39	89.8
910	13.75	200	100	2.8	303	305	109	2747	710	9.6	2.2	11.1	99.9
910	18.25	200	100	2.0	148	51.9	20.9	1454	295	31.2	9.8	1.42	39.8
910	6	200	100	2.5	195	50.7	20.3	849	237	18.3	5.1	4.23	70.8
910	2.5	200	100	3.0	199	34.2	16.4	490	177	15.6	3.8	6.84	98.0
910	1.5	200	100	3.5	254	32.4	19.5	408	168	14.8	4.9	10.8	136
910	0.5	200	100	4.0	286	15.2	5.6	200	66	13.6	3.2	15.2	200
910	0.75	200	100	4.4	246	27.6	11.5	346	109	13.3	2.8	18.4	231
910	0.5	200	100	5.0	297	22.9	11.8	285	120	13.3	3.1	22.9	285
910	8.5	210	90	2.0	404	72.5	33.9	1126	329	17.9	6.8	4.26	66.2
910	4	215	85	2.0	267	40.4	19.6	680	214	19.3	6.3	5.05	85.0
910	3	220	80	2.0	276	38.9	20.4	590	195	17.7	6.3	6.48	98.3
910	2	225	75	2.0	309	39.2	18.3	571	177	16.3	5.1	9.80	143
910	1	230	70	2.0	289	24.2	12.4	336	111	15.9	5.6	12.1	168
910	0.75	235	65	2.0	334	26.6	15.4	308	126	13.2	4.4	17.7	205
930	1	200	100	2.8	377	37.1	18.6	589	181	18.4	6.7	18.6	295
925	2	200	100	2.8	345	76.7	42.9	854	343	13.7	6.5	19.2	214
917	2.5	200	100	2.8	259	28.2	14.6	564	187	23.0	8.1	5.64	113
903	7.25	200	100	2.8	306	48.3	21.7	886	235	20.8	6.8	3.33	61.1
895	10	200	100	2.8	255	33.8	16.9	665	239	22.0	7.2	1.69	33.3
860	6	200	50	1.6									
895	23.5	200	100	2.7									
910	1.7	200	50	1.0									
910	5	200	50	1.0									
910	10.5	200	50	1.0									
925	1	200	50	1.0									
935	5	200	50	1.0	Growth conditions in which nanoribbons are observed but *w*, *l*, *a.r.*, R_w, and R_l are not characterized like above.								
910	1	200	40	1.0									
910	3.3	200	60	1.0									
910	14.5	200	60	1.0									
910	8	200	70	1.0									
910	4	200	70	1.8									
910	5	200	80	2.2									
910	11.75	200	80	2.2									
910	4	200	100	3.2									

Supplementary Discussion

Scanning tunneling spectroscopy (STS) of graphene nanoribbons on Ge(001). Normalized differential conductance as a function of applied bias is measured to probe the electronic density of states (DOS) of 9 nanoribbons with *w* ranging from 5 nm to 19 nm. A representative tunneling spectrum is shown in Supplementary Fig. 8a for a ribbon with *w* of 10 nm (red line) and for a continuous graphene monolayer on Ge(001) (blue line). Compared to a continuous graphene monolayer, the graphene nanoribbons generally have suppressed DOS near the Fermi level and band edges potentially develop, which is consistent with previous tunneling spectra from semiconducting graphene nanostructures[4-7]. We analyze each STS spectrum to estimate a bandgap by using protocols that have been previously established in literature, in which the band edges are linearly extrapolated to the conductance minimum to find the gap[4]. In more detail, the normalized differential conductance (black dots in Supplementary Fig. 8b) is first fit with a 15th order polynomial (green line in Supplementary Fig. 8b) to reduce noise. Then, the minimum of this polynomial is found and a horizontal line that passes through the minimum is defined as the minimum tunneling conductance (blue dashed line in Supplementary Fig. 8b). Another horizontal line is defined 0.1 units above the minimum tunneling conductance (orange dashed line in Supplementary Fig. 8b) and its intersections with the polynomial fit are found. The tangent lines at these intersection points define the band edges (red lines in Supplementary Fig. 8b). The difference in the intersections of the band edges with the minimum tunneling conductance gives the bandgap. Using this technique to analyze all 9 nanoribbons (Supplementary Fig. 9), we find that the bandgap generally varies inversely with *w* (Supplementary Fig. 8c). Gaps as small as 120 meV and as large as 530 meV are measured for ribbons with *w* of 14.5 nm and 7.1 nm, respectively. While it is difficult to make conclusive deductions about the bandgaps from the tunneling spectra due to thermal broadening and tunneling into the underlying substrate[8,9], the spectra are consistent with those previously reported for semiconducting graphene nanostructures[4-7].

Variability in graphene nanoribbon charge transport data. The variability in the on-state conductance and transconductance data in Fig. 5b,c in the main text is likely caused by contact-length variability, which increases as the ribbons become narrower and, thus, shorter. A contact-length on the order of 100 nm is needed to make an ideal contact to graphene[10-12]. However, substantially shorter contact-lengths are possible in our devices depending on the position of the ribbon in the channel, which will statistically vary from device to device. For example, a ribbon with *w* of 10 nm and *l* of 250 nm that is centered in a 160 nm channel is contacted by 45 nm of Pd by both the source and the drain electrode. However, if this ribbon is shifted 30 nm towards the drain, then the drain contact-length increases to 75 nm and the source contact-length decreases to 15 nm, increasing the resistance of the source contact.

Surface morphology after annealing and after nanoribbon growth. Before CH_4 is introduced to initiate nanoribbon synthesis, the Ge substrates are annealed at the growth temperature. During this pre-anneal, the topography and terracing of the Ge(001) surface evolve to yield a gradually rolling morphology. For example, annealing at 910 °C for 0.5 h results in a rolling morphology with periodicity between 2 and 9 μm and amplitude between 0.5 and 8 nm (Supplementary Fig. 12). After CH_4 is introduced, nanoribbons nucleate anywhere on this rolling morphology, near low points (Supplementary Fig. 13c, white "o" symbols), near high points (Supplementary Fig. 13c, yellow "x" symbols), and on sloped regions (Supplementary Fig. 13c, blue "#" symbols). Thus, nucleation does not seem to be strongly correlated with the density or directionality of steps that exist prior to nucleation. Moreover, the nanoribbons are not preferentially aligned with respect to this topography.

Interestingly, after nucleation, local depressions form at the ends of the nanoribbons and the centers of the ribbons become relatively elevated (Supplementary Fig. 14). The slope of these depressions is < 3˚ and typically < 1˚. These depressions are exclusively located where nanoribbons nucleate (Supplementary Fig. 13-14) and do not form during the pre-anneal (Supplementary Fig. 12). Some ribbons have only one end in a depression (Supplementary Fig. 14a, blue "#" symbols), while other ribbons have both ends in

depressions (Supplementary Fig. 14a,c, green “*” symbols). The depressions become wider and deeper as the nanoribbons grow longer and wider. Eventually, in some cases, an entire ribbon can become located in a depression, particularly when the ribbon grows especially wide or when multiple ribbons are located near each other (Supplementary Fig. 14e, white “o” symbols). Depressions are often not observed at the ends of the narrowest ribbons (Supplementary Fig. 14a,c,e, yellow “x” symbols). These observations indicate that the depressions only begin to form after the ribbons nucleate. It is likely that the formation of these depressions is driven by factors similar to those that cause the Ge surface to form nanofacets underneath the ribbons (Supplementary Fig. 14g-i).

Thus, overall, there is no evidence to indicate that pre-existing surface features template the shape, width, or length of the ribbons.

Supplementary Methods

X-ray reflectivity (XRR) studies of the faceted Ge surface. In order to investigate the faceting of the Ge(001) surface underneath graphene, XRR studies are performed on a continuous graphene film grown at 910 °C with x_{CH4} of 0.015 and x_{H2} of 0.33 for 12 h. The facets produce specular x-ray reflections that are interpreted using geometric optics. Similar methods have been used to study the faceting of Si surfaces[13,14]. These measurements provide values for the average facet angle and the upper limit on the distribution of facet angles. They also show that the structure and symmetry exhibited locally (see Fig. 6a-c in the main text) extend across macroscopic areas of the sample. Specifically, the reflectivity data indicate the presence of a four-fold symmetric hill-and-valley surface with facet angles of ~8°. When the substrate is rotated about its surface normal, four intensity maxima with roughly the same magnitude are observed along the Ge<100> directions, indicating that the four facets are uniformly distributed throughout the sample.

Supplementary References

1. Graf, D. et al. Spatially resolved raman spectroscopy of single- and few-layer graphene. *Nano Lett.* **7,** 238-242 (2007).
2. Cancado, L. G., Pimenta, M. A., Neves, B. R. A., Dantas, M. S. S. & Jorio, A. Influence of the atomic structure on the Raman spectra of graphite edges. *Phys. Rev. Lett.* **93,** 247401 (2004).
3. Roy, S. S. & Arnold, M. S. Improving graphene diffusion barriers via stacking multiple layers and grain size engineering. *Adv. Funct. Mater.* **23,** 3638-3644 (2013).
4. Ritter, K. A. & Lyding, J. W. The influence of edge structure on the electronic properties of graphene quantum dots and nanoribbons. *Nat. Mater.* **8,** 235-242 (2009).
5. Magda, G. Z. *et al.* Room-temperature magnetic order on zigzag edges of narrow graphene nanoribbons. *Nature* **514,** 608-611 (2014).
6. Huang, H. *et al*. Spatially resolved electronic structures of atomically precise armchair graphene nanoribbons. *Sci. Rep.* **2,** 983 (2012).
7. Chen, Y. C. *et al*. Tuning the band gap of graphene nanoribbons synthesized from molecular precursors. *ACS Nano* **7,** 6123-6128 (2013).
8. He, K. T., Koepke, J. C., Barraza-Lopez, S. & Lyding, J. W. Separation-dependent electronic transparency of monolayer graphene membranes on III-V semiconductor substrates. *Nano Lett.* **10,** 3446-3452 (2010).
9. Rutter, G. M. *et al.* Imaging the interface of epitaxial graphene with silicon carbide via scanning tunneling microscopy. *Phys. Rev. B* **76,** 235416 (2007).
10. Xia, F. N., Perebeinos, V., Lin, Y. M., Wu, Y. Q. & Avouris, P. The origins and limits of metal-graphene junction resistance. *Nat. Nanotechnol.* **6**, 179-184 (2011).
11. Grosse, K. L., Bae, M. H., Lian, F. F., Pop, E. & King, W. P. Nanoscale Joule heating, Peltier cooling and current crowding at graphene-metal contacts. *Nat. Nanotechnol.* **6**, 287-290 (2011).

12. Huang, B. C., Zhang, M., Wang, Y. J. & Woo, J. Contact resistance in top-gated graphene field-effect transistors. *Appl. Phys. Lett.* **99**, 032107 (2011).

13. Song, S., Yoon, M. & Mochrie, S. G. J. Faceting, tricriticality, and attractive interactions between steps in the orientational phase diagram of silicon surfaces between [113] and [55 12]. *Surf. Sci.* **334,** 153-169 (1995).

14. Song, S. & Mochrie, S. G. J. Attractive step-step interactions, tricriticality, and faceting in the orientational phase diagram of silicon surfaces between [113] and [114]. *Phys. Rev. B* **51,** 10068-10084 (1995).
